# Miscellaneous

**Published:** 1857-12

**Authors:** 


### MISCELLANEOUS.

*A New Luxury.*—A tablespoonful of *Gail Borden's Condensed Milk*, in a cup of coffee or tea, makes a most delicious drink. Those who will try it and learn how to manage it, will never be willing to return to the milk carts, because it is milk in all its purity from farm-house cows.

*Musical.*—What loving father could make a more durable, and elevating, and refining New Year's Gift to a dutiful daughter, than one of *Worcester's* Pianos, whose chief characteristics are, lastingness, harmony, and an aristocratic plainness and richness combined.

CARPETS certainly help to keep a room warm, especially in those many houses in New York, whose cellar ceilings are not plastered. Mr. Landon, 374 Hudson street, will supply our readers on the lowest living terms.

